# Anode‐Less Sulfide‐Based All‐Solid‐State Batteries: Interfacial Challenges, Material Strategies, and Future Prospects

**DOI:** 10.1002/smll.202510624

**Published:** 2025-10-14

**Authors:** Mamta Sham Lal, Paul Albertus, Malachi Noked

**Affiliations:** ^1^ Department of Chemistry Bar‐Ilan University Ramat Gan 529002 Israel; ^2^ Bar‐Ilan Institute of Nanotechnology and Advanced Materials Ramat Gan 529002 Israel; ^3^ Department of Chemical and Biomolecular Engineering A. James Clark School of Engineering University of Maryland College Park MD 20742 USA; ^4^ INIES‐ Israel National Institute for Energy Storage Ramat Gan 5290002 Israel

**Keywords:** Anode‐less solid‐state batteries, Dendrite growth, Li reservoir, Lithium inventory loss, sulfide solid electrolyte

## Abstract

Anode‐less sulfide‐based all‐solid‐state batteries (ASSBs) have emerged as promising candidates for next‐generation energy storage, offering high energy density, enhanced safety, and simplified cell design. By eliminating excess lithium (Li) metal and relying solely on Li extracted from the cathode, these systems significantly improve gravimetric and volumetric performance. However, the absence of a Li reservoir introduces critical challenges, particularly at the Li|solid electrolyte (Li|SE) interface. This review first outlines the fundamental interfacial and electrochemical challenges in anode‐less sulfide systems, including unstable Li plating/stripping, void formation, interfacial contact loss, and parasitic reactions that lead to poor reversibility and early failure. Drawing from recent experimental studies, the second part of this review discusses material and structural strategies developed to stabilize these systems. These include current collector modifications, lithiophilic and alloying interlayers, cathode prelithiation, and artificial interphase engineering, each aiming to suppress dendrite growth, enhance interfacial integrity, and manage Li inventory. The review concludes by highlighting future research directions and design principles essential for realizing scalable and commercially viable anode‐less sulfide‐based ASSBs. By critically evaluating the latest progress, this work aims to provide a comprehensive framework to guide the rational development of robust and high‐performance solid‐state battery architecture.

## Graphical abstract

This review outlines the challenges and strategies for anode‐less sulfide‐based all‐solid‐state batteries, which offer high energy density and safer operation by removing excess lithium. It emphasizes interfacial issues such as void formation and unstable Li plating. It covers approaches like lithiophilic interlayers, cathode prelithiation, and artificial interphases to stabilize interfaces and enhance reversibility, guiding future high‐performance solid‐state battery development.

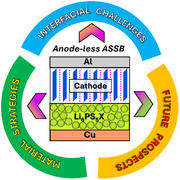

## 1 Introduction

In the pursuit of safer and higher energy density storage systems, all‐solid‐state batteries (ASSBs) incorporating inorganic solid electrolytes (SEs) have gained significant attention as a viable alternative to conventional lithium‐ion batteries.^[^ ^1^, ^2^, ^3^, ^4^ ^]^ Among the diverse classes of SEs, sulfide‐based materials, such as Li_6_PS_5_X (X = Cl, Br, I), Li_10_GeP_2_S_12_, Li_3_PS_4_, have emerged as leading candidates due to their high ionic conductivity, mechanical compliance, and electrochemical stability.^[^ ^5^, ^6^, ^7^, ^8^ ^]^ Their room‐temperature ionic conductivity often matches or surpasses that of organic liquid electrolytes, enabling high‐performance ASSBs without the challenges associated with liquid electrolytes.^[^ ^9^, ^10^, ^11^ ^]^ Moreover, sulfide SEs allow room‐temperature densification and intimate interfacial electrode‐electrolyte contact, avoiding the high sintering temperatures typically required for oxide‐based SEs.^[^ ^12^, ^13^, ^14^ ^]^ Maximizing the energy density of sulfide‐based ASSBs relies on the use of lithium (Li) metal anodes, which offer a theoretical capacity of 3860 mA h g^−1^.^[^ ^15^, ^16^ ^]^ In these systems, the SE functions both as an ion conductor and as a mechanical barrier to suppress Li dendrite growth, motivating extensive research into stable Li metal‐based ASSBs.^[^ ^17^, ^18^ ^]^ Despite advances in interface engineering, such as Li surface treatments and tailored electrolyte formulations, maintaining long‐term interfacial stability remains a critical challenge.^[^ ^19^, ^20^, ^21^ ^]^ This instability stems from the inherent chemical reactivity between Li and sulfide electrolytes, which can lead to uncontrolled interfacial degradation.^[^ ^22^, ^23^ ^]^ Additionally, the soft and deformable nature of sulfides allows dendritic Li to infiltrate the electrolyte, compromising cycling stability and Coulombic efficiency.^[^ ^24^, ^25^ ^]^

**Figure 1:**
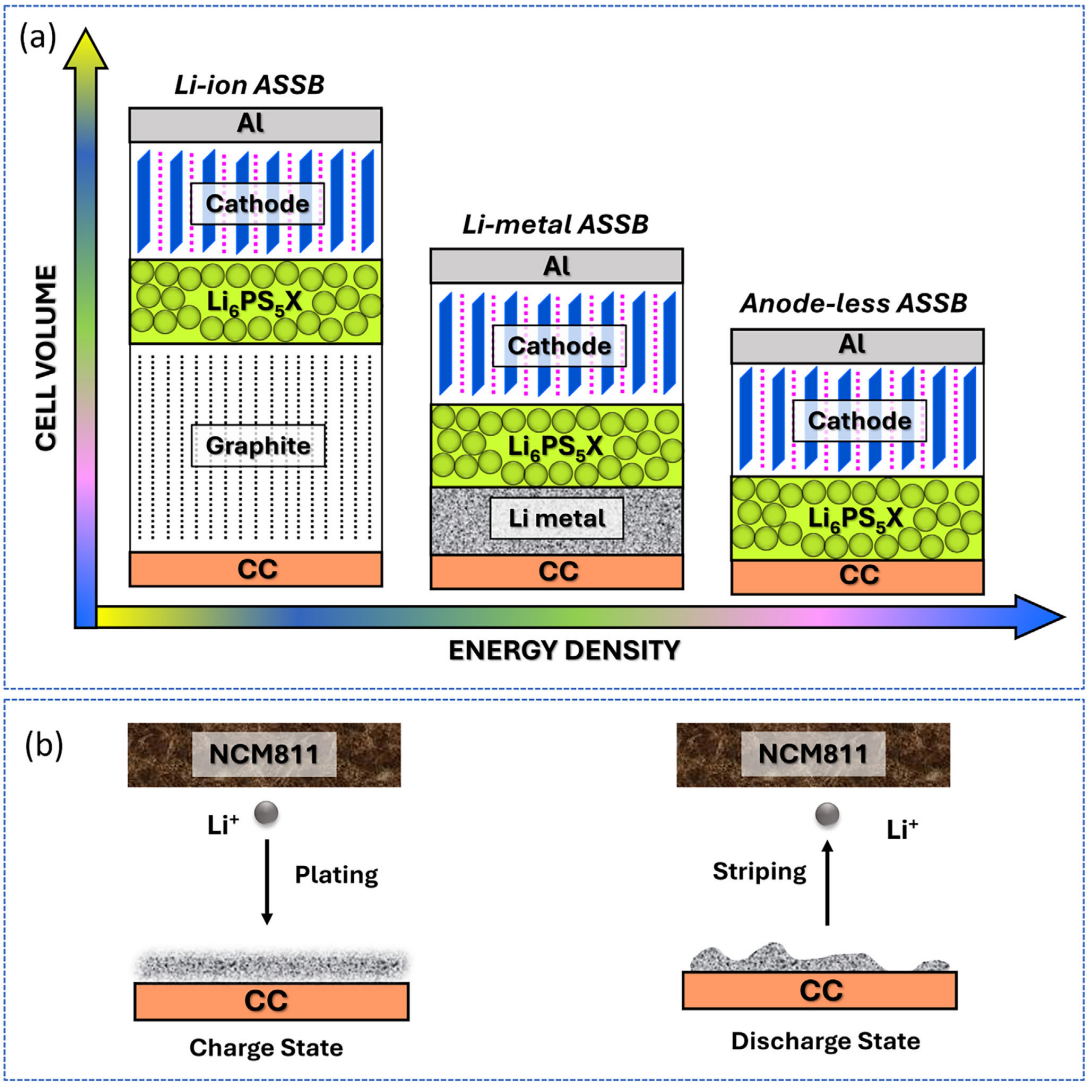
a) Schematic comparison of Li‐ion, Li‐metal, and anode‐less ASSBs. b) Working mechanism of an anode‐less ASSB, where Li is plated onto the current collector during the initial charge.

To address challenges such as dendrite formation and interfacial instability, anode‐less ASSBs have emerged as a promising alternative to traditional Li‐metal ASSBs.^[^ ^26^, ^27^, ^28^ ^]^ In this architecture, the cell is assembled without pre‐deposited Li; instead, with a bare current collector (CC) (typically copper (Cu)) serving as the initial anode. During the first charge, Li‐ions are extracted from the cathode and electrochemically deposited onto the current collector. This plated Li is then stripped during discharge and reinserted into the cathode, enabling reversible cycling.^[^ ^29^, ^30^, ^31^ ^]^ This design offers several advantages: i) Eliminating excess Li reduces inactive mass and volume, increasing both gravimetric and volumetric energy densities.^[^ ^32^ ^]^ ii) It avoids handling ultrathin Li foils, simplifying fabrication, lowering production costs, and improving scalability.^[^ ^33^ ^]^ iii) It inherently improves safety during large‐scale production. Consequently, the anode‐less approach has garnered growing attention as a practical path toward high‐energy‐density ASSBs.^[^ ^34^ ^]^ **Figure** **1**a presents a schematic comparison of Li‐ion, Li‐metal, and anode‐less ASSBs, while Figure 1b illustrates the working mechanism of anode‐less ASSBs.

Despite the promise of anode‐less ASSBs, sulfide‐based systems continue to face critical challenges that hinder practical deployment. A major obstacle is the non‐uniform deposition of Li on the bare metal current collector, which can result in filamentary growth, the formation of electrically isolated “dead” Li, and rapid capacity fading.^[^ ^35^ ^]^ 1) Non‐uniform Li plating and stripping compromise electrochemical efficiency and accelerate dendrite formation, posing serious safety and performance risks.^[^ ^36^ ^]^ 2) Chemical incompatibility between plated Li and sulfide SEs further aggravates interfacial degradation, leading to increased internal resistance and structural breakdown of the SE during cycling.^[^ ^37^ ^]^ 3) The inherently limited Li inventory in anode‐less ASSBs, unlike Li‐excess systems, offers no buffer against Li loss, thereby constraining long‐term cycling stability and overall battery reliability.^[^ ^38^ ^]^ To overcome these interconnected issues, several engineering strategies have been explored, including modifying the current collector to regulate Li nucleation, incorporating artificial or reactive interlayers to stabilize interfaces, implementing cathode prelithiation to compensate for Li loss, and tailoring solid electrolyte interphases (SEIs) to enhance chemical compatibility.^[^ ^39^, ^40^ ^]^ For instance, Lee et al. developed an anode‐less solid‐state Li battery using an In‐doped Ag‐coated current collector where the alloying reaction between Li and Ag‐In interlayer enhanced interfacial stability, enabling over 250 stable cycles with a Coulombic efficiency of 99.8%.^[^ ^26^ ^]^ Similarly, Gu et al. systematically investigated the influence of current collector surface roughness on Li plating/stripping behavior, demonstrating that a moderately roughened surface enhances Coulombic efficiency and cycling stability by improving interfacial contact with the sulfide SE, while excessive roughness degrades performance.^[^ ^41^ ^]^ Collectively, these approaches aim to unlock the full potential of sulfide‐based anode‐less ASSBs, offering pathways toward high‐performance and scalable energy storage systems.

While evaluating studies in this field, it is important to consider several key performance metrics that directly influence the interpretation of reported results. These include the initial capacity of Li plated, the amount of Li plated and stripped in each cycle, the stack pressure applied during initial plating and subsequent cycling, and the current density during both initial and later cycles.^[^ ^29^, ^42^, ^43^ ^]^ The presence and thickness of interlayers, relative to the amount of Li cycled, as well as the source of Li (pre‐deposited foil or Li supplied by the cathode), are also critical factors.^[^ ^44^ ^]^ In particular, the areal capacity per cycle can strongly affect cycling stability: cycling with very thin Li layers (< a few microns) may appear stable over many cycles, whereas thicker Li layers (>10 µm, relevant for commercial cells) often lead to faster degradation.^[^ ^33^ ^]^ For context, commercially relevant cathode loadings are typically ≥ 3 mAh cm^−2^, corresponding to ≈15 µm of 100% dense Li.^[^ ^45^ ^]^ Thus, studies reporting long cycle life at very low areal capacity should be interpreted differently from those demonstrating fewer cycles at higher, commercially relevant capacities, highlighting the need to contextualize performance metrics when comparing studies. In recent years, significant progress has been made toward achieving commercially relevant cathode loadings (≥ 3 mAh cm^−2^) in sulfide‐based ASSBs.^[^ ^46^ ^]^ High‐loading cathodes have been realized using strategies such as optimizing composite cathode microstructure, incorporating conductive additives, and employing protective interlayers or surface coatings to improve Li^+^ transport and mitigate interfacial degradation.^[^ ^47^ ^]^ Despite these advances, challenges remain: thick cathodes can induce higher interface resistance, mechanical stress, and uneven Li deposition, which compromise cycling stability.^[^ ^48^ ^]^ These observations underscore the importance of combining electrode engineering, interface design, and optimized cycling protocols to achieve high‐energy‐density, stable, and scalable anode‐less sulfide‐based ASSBs.

Although interest in anode‐less configurations within ASSB research has grown rapidly, dedicated reviews focusing specifically on sulfide‐based anode‐less ASSBs remain limited. With the surge in experimental and theoretical efforts in this emerging field, a timely and comprehensive review is necessary to consolidate recent developments, critically assess ongoing challenges, and highlight future research directions. This article aims to fill that gap by providing an in‐depth overview of the current status of anode‐less sulfide‐based ASSBs. This review begins by outlining the fundamental challenges, such as interfacial instability, lithium inventory loss, and non‐uniform Li plating, that hinder their practical realization. It then categorizes and discusses recent strategies aimed at addressing these issues, including current collector engineering, interlayer design, cathode prelithiation, and SEI modifications. Finally, we conclude with a forward‐looking discussion on the critical research gaps and opportunities that could pave the way for the scalable development of high‐performance sulfide‐based anode‐less ASSBs.

## 2 Interfacial and Electrochemical Challenges in Anode‐Less Sulfide Systems

The pursuit of high‐energy, safe, and scalable battery systems has brought anode‐less sulfide‐based ASSBs to the forefront of next‐generation energy storage research. By removing the excess Li metal from the anode and relying solely on Li extracted from the cathode during the initial charge, these systems offer remarkable advantages, including improved gravimetric and volumetric energy densities, enhanced safety, and simplified manufacturing. However, these benefits are counterbalanced by critical interfacial and electrochemical challenges that severely impact long‐term performance. Chief among these is the instability of the Li‐SE interface, non‐uniform Li plating/stripping, and irreversible Li loss due to the absence of a Li reservoir. The high reactivity between plated Li and sulfide SEs further exacerbates interfacial degradation, resulting in increased resistance and morphological breakdown throughout cycling.

**Figure 2:**
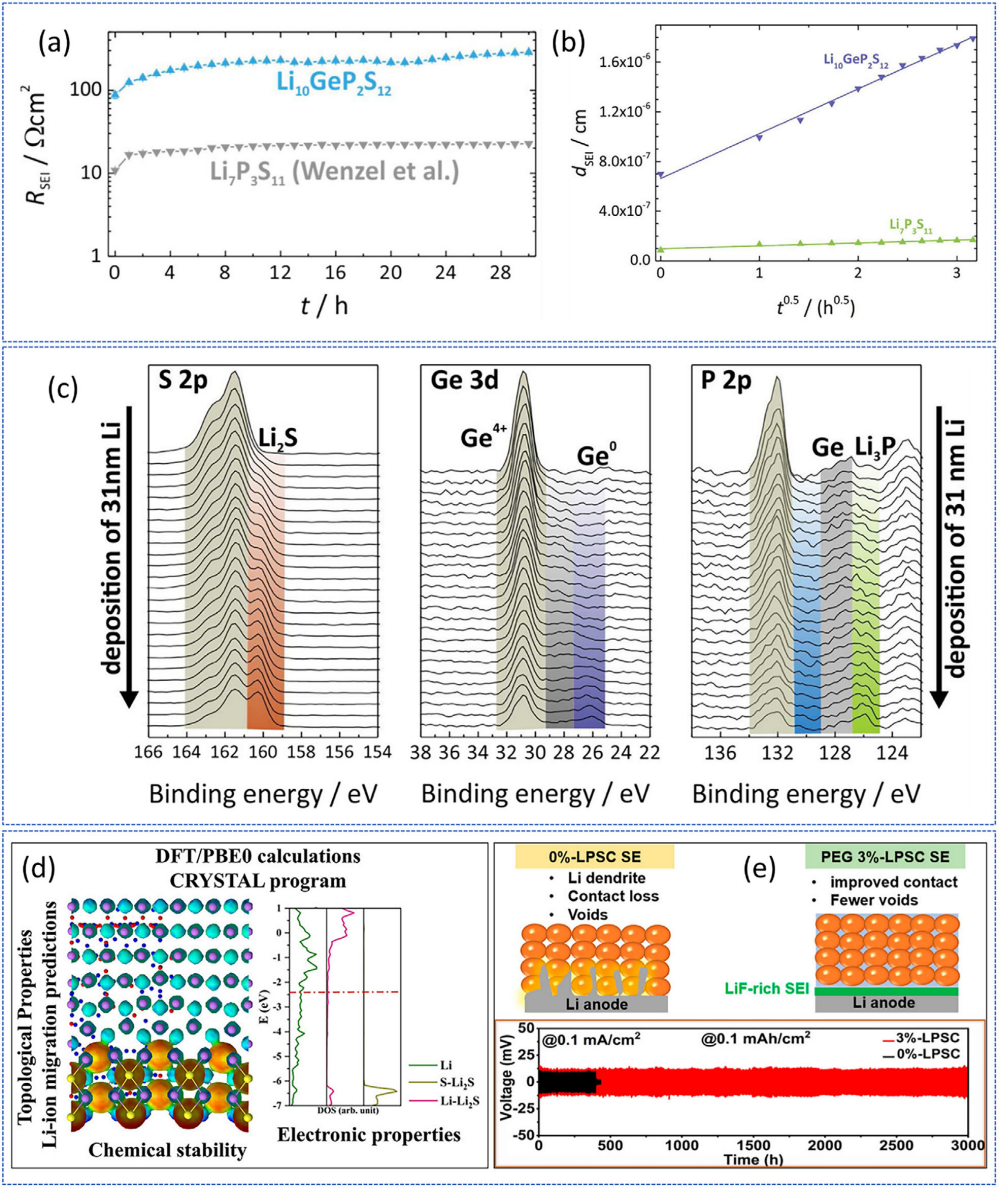
a) Comparison of Li_10_GeP_2_S_12_/Li and Li_7_P_3_S_11_/Li interfacial resistances, showing higher resistance for Li_10_GeP_2_S_12_/Li. b) SEI thickness as a function of the square root of time, indicating parabolic growth consistent with a diffusion‐controlled mechanism. c) Evolution of S 2p, Ge 3d, and P 2p/Ge 3p XPS spectra during Li (31 nm) deposition on Li_10_GeP_2_S_12_, indicating decomposition and formation of new species. Adapted with permission.^[^ ^49^ ^]^ Copyright 2016, American Chemical Society. d) DFT/PBE0 (CRYSTAL) workflow for sulfide SEs. From left to right: topology‐guided Li‐ion pathway predictions, interfacial chemical‐stability visualization, and calculated DOS highlighting electronic structure near the Fermi energy. Reproduced under the terms of the CC‐BY 4.0 license.^[^ ^51^ ^]^ Copyright 2023, The Authors. e) Effect of PEG additive on LPSC‐Li interface and cycling. Top: schematic contrasting pristine LPSC with PEG 3% LPSC, which forms a LiF‐rich SEI and improves interfacial contact. Bottom: Symmetric Li|SE|Li cycling shows stable polarization for PEG‐3% LPSC. Reproduced under the terms of the CC‐BY 4.0 license.^[^ ^52^ ^]^ Copyright 2024, American Chemical Society.

A comprehensive understanding of the chemical and kinetic nature of the Li‐SE interface is crucial for identifying the origins of failure. For instance, Wenzel et al. investigated the interfacial behavior of Li_10_GeP_2_S_12_ (LGPS) in contact with Li metal using time‐resolved impedance spectroscopy and in situ X‐ray photoelectron spectroscopy.^[^ ^49^ ^]^ Although LGPS and Li_7_P_3_S_11_ share similar tetrahedral PS_4_ structural motifs, the presence of Ge^4+^ in LGPS, a more easily reducible cation compared to P^5+^, leads to significantly faster interfacial decomposition. The resulting SEI comprises Li_2_S, Li_3_P, and a Ge‐containing phase (elemental Ge or Li‐Ge alloy), causing a substantial increase in interfacial resistance (R_SEI_ ≈ 250 Ω·cm^2^ after 24 h, compared to the bulk LGPS resistance of ≈60 Ω·cm^2^). SEI growth follows a diffusion‐controlled, parabolic rate law, resulting in an estimated thickness of ≈370 nm after one year and a corresponding interphase resistance of ≈4.6 kΩ·cm^2^, whereas Ge‐free Li_7_P_3_S_11_ exhibits considerably lower interfacial resistance and thinner SEI (≈23 nm, R_SEI_ ≈ 0.28 kΩ·cm^2^) (**Figure** **2**a–c). These results demonstrate that both the crystal structure and elemental chemistry of sulfide SEs significantly influence interfacial reaction kinetics, offering insight into the fundamental origins of interfacial instability in anode‐less sulfide‐based ASSBs. Complementing the experimental observations, first‐principles calculations by Hao et al. indicate that lithiation of Li_3_PS_4_ (LPS) and Li_10_GeP_2_S_12_ (LGPS) is thermodynamically favorable, with PS_4_^3‐^ and GeS_4_^4−^ polyanions gradually disintegrating into S^2−^, P^3−^, and Ge^4−^ anions.^[^ ^50^ ^]^ While both materials retain Li‐ion transport and electronic‐blocking properties upon partial lithiation, LGPS undergoes faster structural changes due to the presence of Ge, whereas LPS shows transient P cluster formation. Additionally, densification of the lithiated phases may contribute to void formation at the interface, providing further insight into the subtle differences in reactivity between LGPS and LPS.

Building upon these insights, theoretical work on the Li_3_PS_4_/Li (LPS/Li) interface by Marana et al. further clarifies the role of crystal structure and chemistry in interfacial stability.^[^ ^51^ ^]^ At the LPS/Li interface, direct contact with Li metal induces spontaneous redox decomposition: Li_3_PS_4_ reacts to form Li_2_S and Li_3_P, with P^5+^ reduced to P^3−^ and Li oxidized to Li^+^. This reaction occurs despite a negligible lattice mismatch, confirming that chemical composition rather than mechanical factors governs reactivity. The PS_4_ tetrahedra undergo significant structural rearrangements, with P atoms migrating into the Li metal and Li‐S bonds forming a Li_2_S‐like lattice. Topological analyses reveal these bonds retain predominantly ionic character, facilitating Li^+^ transport during SEI growth. In contrast, introducing a Li_2_S layer between LPS and Li forms a stable passivating interface with strong adhesion, low strain, and favorable electronic alignment, thereby preventing further decomposition while maintaining Li^+^ conductivity (Figure 2d). Further extending these findings, Serbessa et al. demonstrated that Li_6_PS_5_Cl (LPSC), when paired with Li through an in situ LiF‐rich SEI generated from a composite electrolyte containing LiFSI and PEG, achieves enhanced interfacial stability.^[^ ^52^ ^]^ The LiF‐rich SEI suppresses decomposition and dendrite formation while simultaneously improving mechanical contact and Li^+^ transport (Figure 2e). Taken together, these comparative studies on LGPS, LPS, and LPSC establish that both intrinsic factors (crystal structure, reducibility of constituent cations) and extrinsic strategies (engineered SEIs) critically govern interfacial reaction kinetics, deepening our understanding of the fundamental origins of instability in sulfide‐based ASSBs.

To explore how mechanical pressure influences Li deposition behavior, Park et al. investigated failure mechanisms in anode‐less ASSBs by examining Li plating onto stainless steel current collectors under varying stack pressures (2–20 MPa) with Li_6_PS_5_Cl (LPSCl) as the SE.^[^ ^53^ ^]^ Their study revealed that uneven interfacial contact at low pressures leads to localized current focusing, which promotes Li filament growth and short‐circuiting. Increasing pressure initially improves interfacial contact and suppresses early failure by promoting more uniform Li nucleation, though cracks can still form at stress‐concentrated protrusions. The intrinsic surface roughness of LPSCl pellets (up to ≈7 µm) causes the applied stack pressure to be distributed non‐uniformly across the interface; protrusions experience higher local stress while valleys remain poorly contacted. This uneven pressure distribution leads to spatially heterogeneous Li nucleation and isolated Li islands at low pressure, whereas high pressures promote more uniform Li coverage, reducing local current hotspots and mitigating Li filament initiation, though some cracks still form due to mechanical stress at notches. Surface profiling of LPSC pellets further confirmed their heterogeneous behavior. These findings highlight the non‐linear role of stack pressure in controlling interfacial uniformity and stability.

To further elucidate short‐circuit mechanisms, Park et al. employed X‐ray computed tomography to visualize Li filament growth and pressure‐induced fractures.^[^ ^53^ ^]^ While pristine SE pellets were crack‐free, electrochemical cycling led to fracture formation that often propagated toward the Li counter electrode, creating a high risk of short‐circuiting. Cracks form due to the combined effects of Li plating stress and stack pressure, with stress concentrated at protrusions and notches. Some cracks were likely filled with Li, while others remained voids, reflecting the stochastic nature of plating‐induced failure. Overall, this study clarifies that moderate stack pressure is beneficial for uniform Li deposition and interfacial contact, while also showing that pressure can have a dual role by inducing stress‐concentrated fractures that contribute to crack formation.

**Figure 3:**
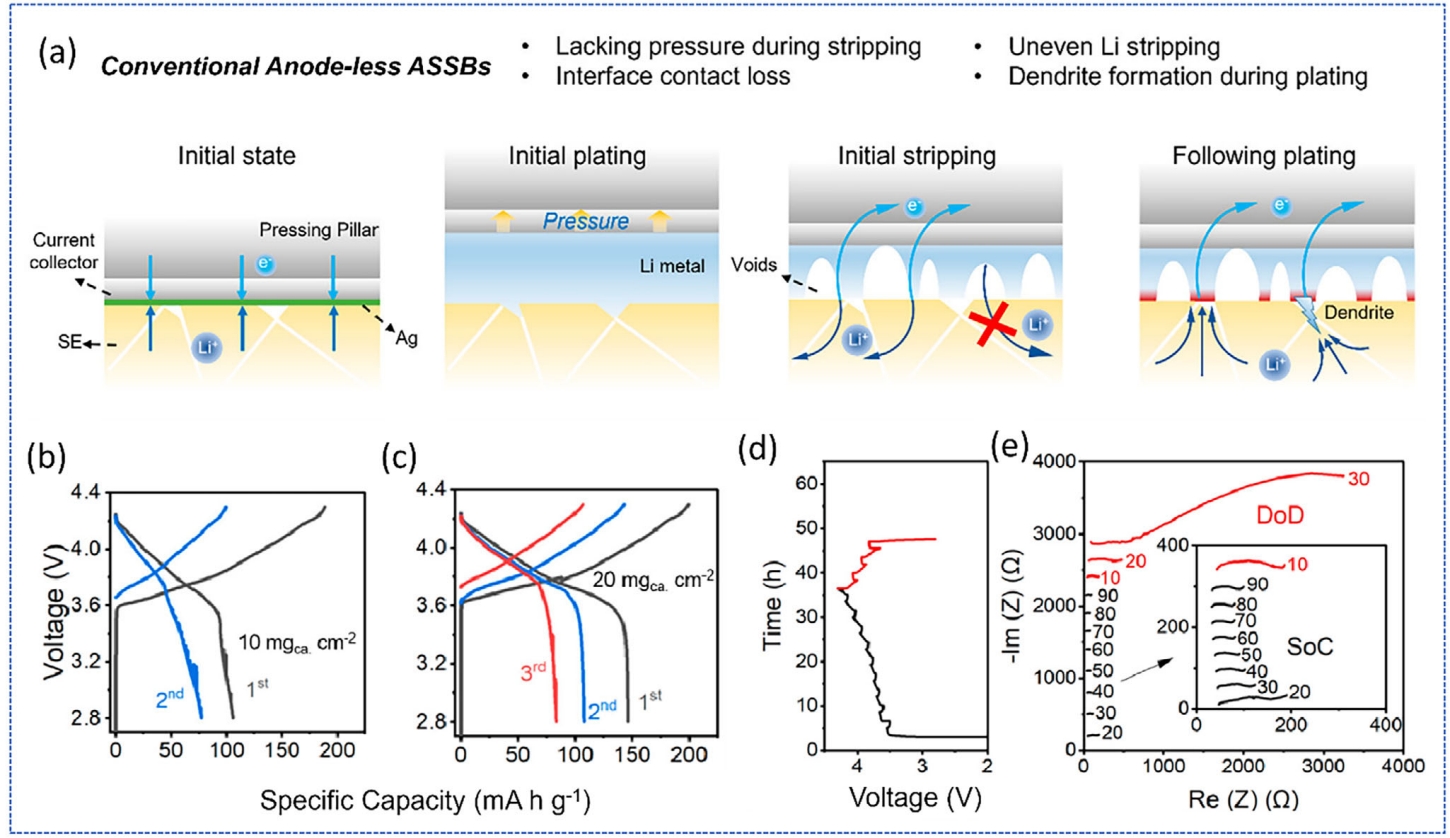
a) Conventional anode‐less ASSBs during the initial state, initial plating, initial stripping, and subsequent plating. Galvanostatic charge–discharge profiles of anode‐less ASSBs (b) at a cathode mass loading of 10 mg cm^−2^ and (c) at a cathode mass loading of 20 mg cm^−2^. d) Charge–discharge profiles of anode‐less ASSBs in the EIS measurement. e) Nyquist plots of anode‐less ASSBs at different SoC and DoD. Adapted under the terms of the CC‐BY 4.0 license.^[^ ^54^ ^]^ Copyright 2023, American Chemical Society.

While effective pressure management is vital, another critical challenge is the loss of interfacial contact during cycling. Cao et al. employed operando techniques to investigate anode‐less sulfide‐based ASSBs, revealing that low initial Coulombic efficiency and rapid capacity fade, particularly at high cathode loadings, stemmed from interfacial contact loss during Li stripping.^[^ ^54^ ^]^ The authors illustrated that in conventional anode‐less ASSBs, where the current collector is directly compressed onto the SE, Li plating induces significant volume expansion, which increases internal pressure and fills surface voids in the SE (**Figure** **3**a). During stripping, Li is preferentially removed from well‐connected interface regions, creating voids at the SE surface. In the absence of sufficient mechanical pressure during this process, residual Li forms a porous, disconnected structure. This leads to low Coulombic efficiency and elevated interfacial impedance. Upon subsequent plating, poor SE‐Li contact results in uneven current and ion flux distribution, ultimately promoting localized dendrite growth.

To further investigate these interfacial limitations, the authors constructed anode‐less sulfide‐based ASSBs using a high‐loading single‐crystal LiNi_0.8_Mn_0.1_Co_0.1_O_2_ (NMC811) cathode paired with a high‐conductivity sulfide electrolyte (Li_5.4_PS_4.4_Cl_1.6_, 7.8 mS cm^−1^), and a 20 nm Ag‐coated stainless steel current collector to enhance lithiophilicity. The cells were tested under an external pressure of 7.5 MPa. As shown in Figure 3b,c for cathode loadings of 10 and 20 mg cm^−2^, respectively, the discharge profiles displayed considerable fluctuations and rapid capacity decline. An initial Coulombic efficiency of only 58.4% was observed at 10 mg cm^−2^, indicating incomplete Li stripping and highlighting the intrinsic reversibility challenges associated with conventional anode‐less sulfide‐based ASSBs. To probe the origin of low stripping efficiency, the authors conducted electrochemical impedance spectroscopy (EIS) at different states of charge using galvanostatic intermittent titration (GITT) at C/20 (Figure 3d,e). A sharp increase in interfacial resistance, exceeding 3000 Ω during discharge, was observed, correlating with the rapid voltage drop and reinforcing the link between contact loss and reversibility in anode‐less sulfide‐based ASSBs.

**Figure 4:**
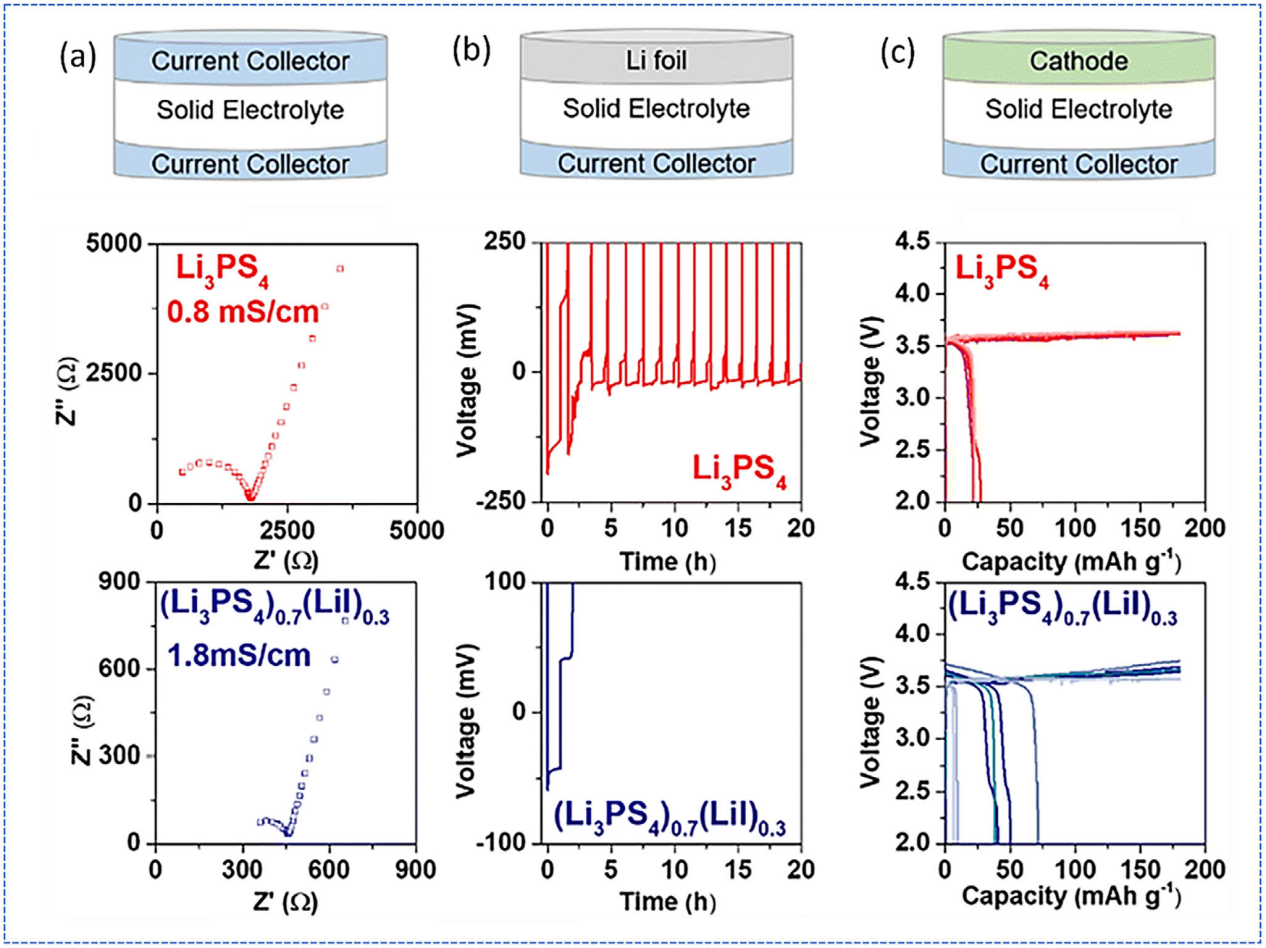
Electrochemical properties of SEs for anode‐less sulfide‐based ASSBs. a) Nyquist plots and the ionic conductivities of the SE layers, b) the Li plating/stripping curves of asymmetric cells under 0.5 mA h cm^−2^, and c) galvanostatic charge–discharge curves of full‐cells under 0.1 C at 60 °C for Li_3_PS_4_ and (Li_3_PS_4_)_0.7_(LiI)_0.3_ (from top to bottom). Adapted with permission.^[^ ^56^ ^]^ Copyright 2023, Royal Society of Chemistry.

In support of these observations, Lewis et al. studied Li plating and stripping behavior in anode‐less ASSBs using Li_6_PS_5_Cl as the SE. Through advanced characterization and modelling, they revealed that local Li depletion during stripping limits cycling stability and promotes filament growth.^[^ ^55^ ^]^ Specifically, non‐uniform Li stripping creates isolated Li islands and voids at the SE interface, which concentrate current at the remaining Li regions. This locally elevated current density accelerates void propagation and provides hotspots for dendritic nucleation during subsequent plating. Their findings emphasize that such localized interfacial instabilities lead to rapid short‐circuiting, rather than gradual capacity fade from limited Li inventory, making short‐circuiting the primary failure mode in these systems. In contrast, pure Li inventory loss typically results in a slow decline in capacity, whereas dendrite‐induced short‐circuits cause sudden and irreversible cell failure, highlighting the dominant role of short‐circuiting in anode‐less sulfide‐based ASSBs. In another comparative study, Gu et al. systematically investigated the impact of different sulfide‐based SEs on Li plating/stripping behavior in anode‐less configurations using asymmetric Li||SS cells.^[^ ^56^ ^]^ Among them, the Li_3_PS_4_‐based cell exhibited extremely poor reversibility, failing after just a single cycle due to rapid short‐circuiting, highlighting its instability under repeated Li deposition and dissolution (**Figure** **4**). Similarly, incorporation of LiI to form a glass‐ceramic (Li_3_PS_4_)_0.7_(LiI)_0.3_ SE did not improve interfacial stability, as the cell ceased functioning after the first cycle. Furthermore, full‐cell cycling without additional stack pressure revealed that glass‐ceramic sulfide SEs exhibited large irreversible capacities, underscoring their unsuitability for anode‐free designs, where efficient and stable Li plating/stripping is critical.

**Figure 5:**
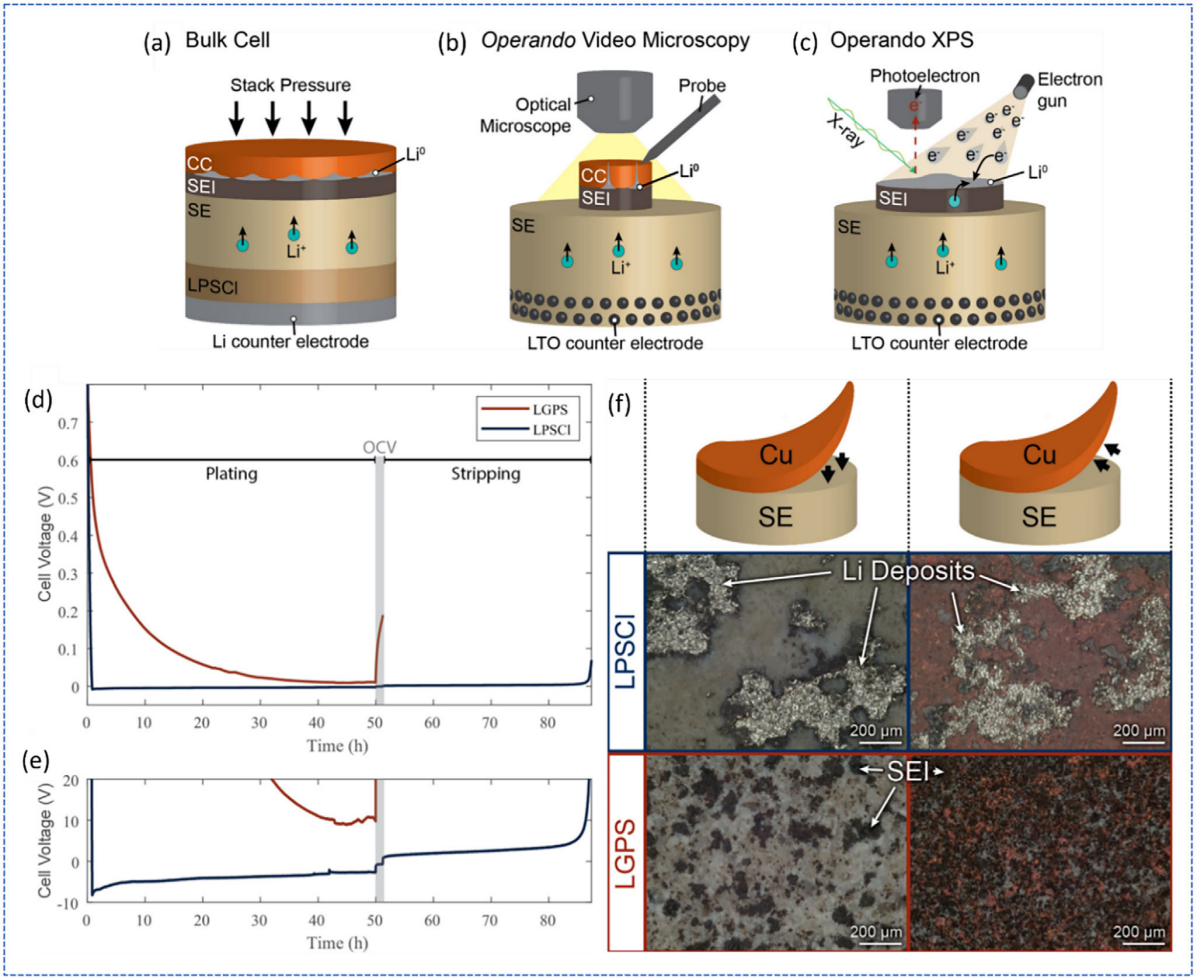
The study used three cell configurations to plate Li onto anode‐free surfaces: a) a bulk cell with a Cu current collector and a protective LPSCl layer when using LGPS; b) an operando video microscopy cell with a sputtered Mo current collector; and c) an operando XPS cell using an electron gun as a virtual electrode. d) Bulk cell voltage profiles at 10 µA cm^−2^ show Li plating behavior; e) Zoomed‐in view reveals a nucleation dip below 0 V in LPSCl, absent in LGPS, indicating suppressed Li plating. f) Optical images after one half‐cycle show metallic Li deposits and minimal SEI on LPSCl, while LGPS displays extensive SEI byproducts and no visible Li, indicating severe interfacial degradation. Adapted with permission.^[^ ^57^ ^]^ Copyright 2021, IOPscience.

To provide chemical‐level insights, Davis et al. investigated the interfacial interactions between Li and sulfide SEs in anode‐free cells using operando XPS and video microscopy.^[^ ^57^ ^]^ They revealed that Li_6_PS_5_Cl (LPSCl) forms an electronically insulating SEI that supports stable Li plating, whereas Li_10_GeP_2_S_12_ (LGPS) forms a conductive interphase that promotes continuous decomposition and hinders plating. Using bulk Li/SE/Cu cells (**Figure** **5**a) at a low current density of 10 µA cm^−2^_,_ the authors captured the initial voltage response and the transition from SE decomposition to Li plating. As shown in Figure 5d,e, a distinct nucleation behavior was observed with LPSCl, where the voltage dropped below 0 V, followed by a nucleation peak, indicating effective Li plating. Postmortem analysis confirmed clean interfaces and the presence of substantial Li deposits (Figure 5f). In contrast, LGPS showed no nucleation features, and the voltage remained above 0 V throughout, suggesting suppressed Li nucleation due to continuous decomposition. Postmortem characterization revealed severe interfacial degradation and thick SEI accumulation (Figure 5f). Operando video microscopy and XPS further confirmed that in LPSCl, initial SEI formation is followed by stable Li deposition, whereas in LGPS, continuous SEI growth dominates without a transition to plating. Together, these results demonstrate the higher Faradaic efficiency and interfacial stability of the Cu/LPSCl interface compared to Cu/LGPS, highlighting the crucial role of the Li‐sulfide interphase in determining the initial plating behavior of anode‐less ASSBs.

**Figure 6:**
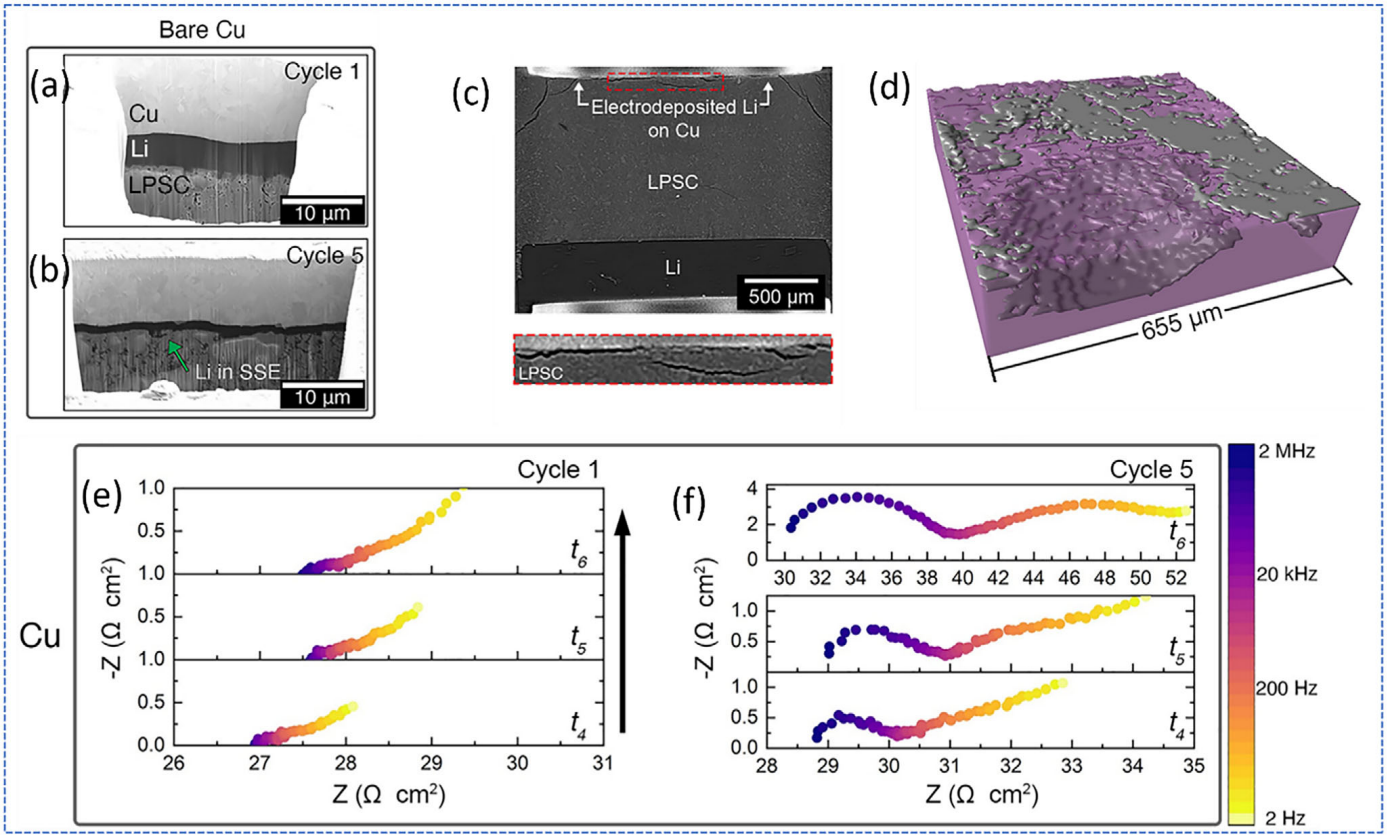
a,b) Cryo‐FIB‐SEM characterization at different stages of cycling. c,d) Synchrotron X‐ray microcomputed tomography characterization of a bare Cu electrode after electrodeposition. e,f) In situ EIS analysis during Li plating/stripping. Adapted with permission.^[^ ^58^ ^]^ Copyright 2023, Elsevier.

On the microstructural side, Sandoval et al. employed focused ion beam (FIB) milling combined with scanning electron microscopy (SEM) to investigate the structural evolution of Li deposited on bare Cu in anode‐less cells.^[^ ^58^ ^]^ To minimize beam‐induced artifacts, both plasma FIB (PFIB) and cryogenic Ga^+^ FIB (cryo‐FIB) techniques were utilized (**Figure** **6**a,b). After a deposition of 1.0 mA h cm^−2^ (theoretically corresponding to 5 µm thickness), PFIB‐SEM imaging revealed that the Li ion on bare Cu was highly non‐uniform and significantly thinner than expected, reaching only ≈3.4 µm at its thickest point. The deposited Li also exhibited internal porosity and extended into the pores of SE. Further cryo‐FIB‐SEM analysis after repeated deposition cycles showed progressively increasing morphological irregularities. By the 5th cycle, the Li layer was notably thin and uneven, with extensive Li penetration into the SE, correlating with short‐circuit failure. These observations underscore the challenges associated with uncontrolled Li growth at the Cu/sulfide SE interface in the absence of interfacial engineering. To investigate the spatial distribution of deposited Li beyond the localized regions accessible by FIB, Sandoval et al. employed synchrotron X‐ray micro‐computed tomography (µCT) to visualize Li growth on bare Cu across larger interfacial areas (Figure 6c,d). After depositing 2 mA h cm^−2^ of Li at 0.5 mA cm^−2^, reconstructed µCT images revealed highly non‐uniform Li deposition with significant thickness variations and evidence of Li penetration into cracks within the SE. 3D renderings confirmed irregular Li coverage and growth within interfacial voids. These volumetric results corroborated the FIB‐SEM findings, reinforcing the conclusion that bare Cu promotes non‐uniform and unstable Li growth in anode‐less sulfide‐based configurations.

To further understand interfacial evolution during cycling, the authors performed in situ potentiostatic EIS measurements on bare Cu electrodes (Figure 6e,f). During the 1st stripping cycle, the spectra remained largely unchanged, suggesting minimal contact degradation at the interface. However, by the 5th stripping cycle, the emergence and growth of distinct high‐ and low‐frequency features indicated increasing interfacial and constriction resistance. This evolution was attributed to progressive contact loss and void formation at the Cu‐Li interface, driven by localized depletion of Li near the end of stripping. These findings highlight the intrinsic vulnerability of bare Cu electrodes in anode‐less sulfide‐based configurations, where interfacial degradation intensifies with continued cycling.

**Figure 7:**
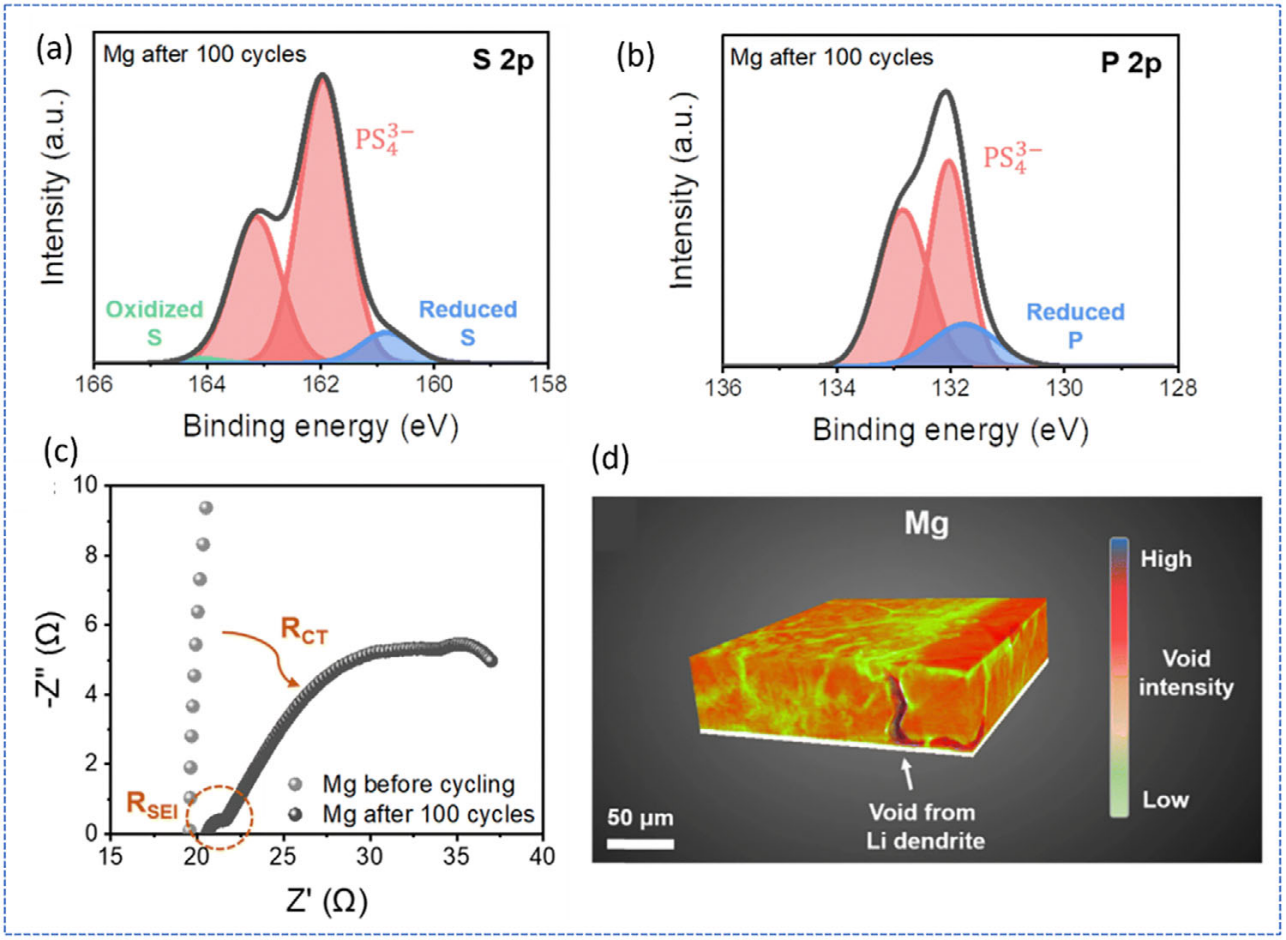
XPS results of the anode‐SE interface after 100 cycles of half‐cell evaluation, a) S 2p and b) P 2p spectra. EIS Nyquist plot of half‐cell after 100 cycles with Mg thin‐film electrode. XRM 3D image of anode‐SE interface after 100 cycles in a half‐cell using Mg thin‐film. Adapted with permission.^[^ ^59^ ^]^ Copyright 2024, Royal Society of Chemistry.

Focusing on long‐term degradation, Oh et al. systematically investigated Li inventory loss mechanisms in anode‐less sulfide‐based ASSBs by examining the interfacial degradation and morphological instability of Li during repeated plating/stripping cycles on bare Mg and bilayer‐protected current collectors.^[^ ^59^ ^]^ Recognizing the vulnerability of anode‐less systems to irreversible Li consumption, due to the absence of excess Li, they emphasized how direct contact between the deposited Li and the sulfide SE Li_6_PS_5_Cl (LPSCl) leads to parasitic side reactions and unstable solid‐electrolyte interphase (SEI) formation. X‐ray photoelectron spectroscopy (XPS) after 100 cycles revealed significant Li_2_S and Li_3_P formation on bare Mg, indicating extensive interfacial reactivity and active Li loss (**Figure** **7**a,b). Electrochemical impedance spectroscopy (EIS) corroborated this by showing increased interfacial resistance linked to SEI buildup and Li filament growth (Figure 7c). Additionally, 3D X‐ray microscopy (XRM) visualizations uncovered void formation and Li penetration into the SE layer over time, confirming morphological instability and progressive Li isolation (Figure 7d). These findings underscore that, without interfacial regulation, bare anode surfaces enable non‐uniform Li deposition, SE degradation, and cumulative Li loss, compromising both capacity retention and safety in anode‐less configurations.

To evaluate performance from a system‐level perspective, Nanda et al. conducted a comprehensive analysis of Li inventory loss in anode‐less full cells, identifying it as the primary factor limiting long‐term cyclability.^[^ ^60^ ^]^ Unlike conventional Li‐ion systems that employ excess Li or host materials like graphite, anode‐free configurations rely solely on Li extracted from the cathode, rendering any loss of Li effectively irreversible. The authors emphasized that the inefficiency in Li plating and stripping, exacerbated by the formation of mossy or dendritic Li, unstable SEI layers, and parasitic side reactions, leads to the progressive isolation of electrochemically inactive “dead” Li. To address this, they introduced the Li inventory retention rate (LIRR) as a more reliable metric than Coulombic efficiency (CE) for comparing performance across systems, especially in practical full‐cell architectures. Through a critical evaluation of strategies, including electrolyte formulation, current collector modification, and cell formation protocols, the study highlighted the importance of achieving highly reversible Li cycling (LIRR ≥ 99.9%) to unlock the potential of anode‐less Li‐metal batteries for commercial applications.

Taken together, these studies highlight the multifaceted nature of interfacial and electrochemical degradation in anode‐less sulfide‐based ASSBs. Their collective insights provide the groundwork for the development of targeted stabilization strategies, which are discussed in detail in the following section.

## 3 Strategies to Stabilize Anode‐Less Sulfide‐Based ASSBs

To address the critical challenges of interfacial instability, Li inventory loss, and non‐uniform Li plating in anode‐less sulfide‐based ASSBs, recent research has focused on strategic material and structural innovations. These include current collector modifications to regulate Li nucleation, interlayer engineering to buffer interface reactions and accommodate volume changes, cathode prelithiation techniques to compensate for Li loss, and tailored SEI modifications to enhance both chemical and mechanical stability. This section reviews these key approaches, highlighting their underlying mechanisms, representative studies, and potential for enabling high‐performance and scalable anode‐less ASSBs.

Wang et al. presented a compelling strategy to stabilize anode‐free sulfide‐based ASSBs by modifying the current collector surface to enhance Li metal wetting and uniform deposition.^[^ ^40^ ^]^ Recognizing the challenge of achieving stable cycling without a Li reservoir, the authors introduced a lithiophilic interfacial layer of Li_2_Te, formed in situ via electrochemical activation of a thermally deposited Cu_2_Te layer on a Cu current collector. This 1 µm‐thick Li_2_Te layer remains structurally stable during cycling and serves as a conductive, wettable scaffold for Li plating and stripping, directly influencing interfacial morphology and electrochemical reversibility. This approach is classified as a “surface modification strategy,” where the current collector is directly coated or treated to create a lithiophilic interface. Its key advantage lies in the simplicity of implementation and direct improvement of Li nucleation. However, such surface modifications primarily affect the 2D interface and may offer limited mitigation against 3D Li growth or large volume changes during cycling.

**Figure 8:**
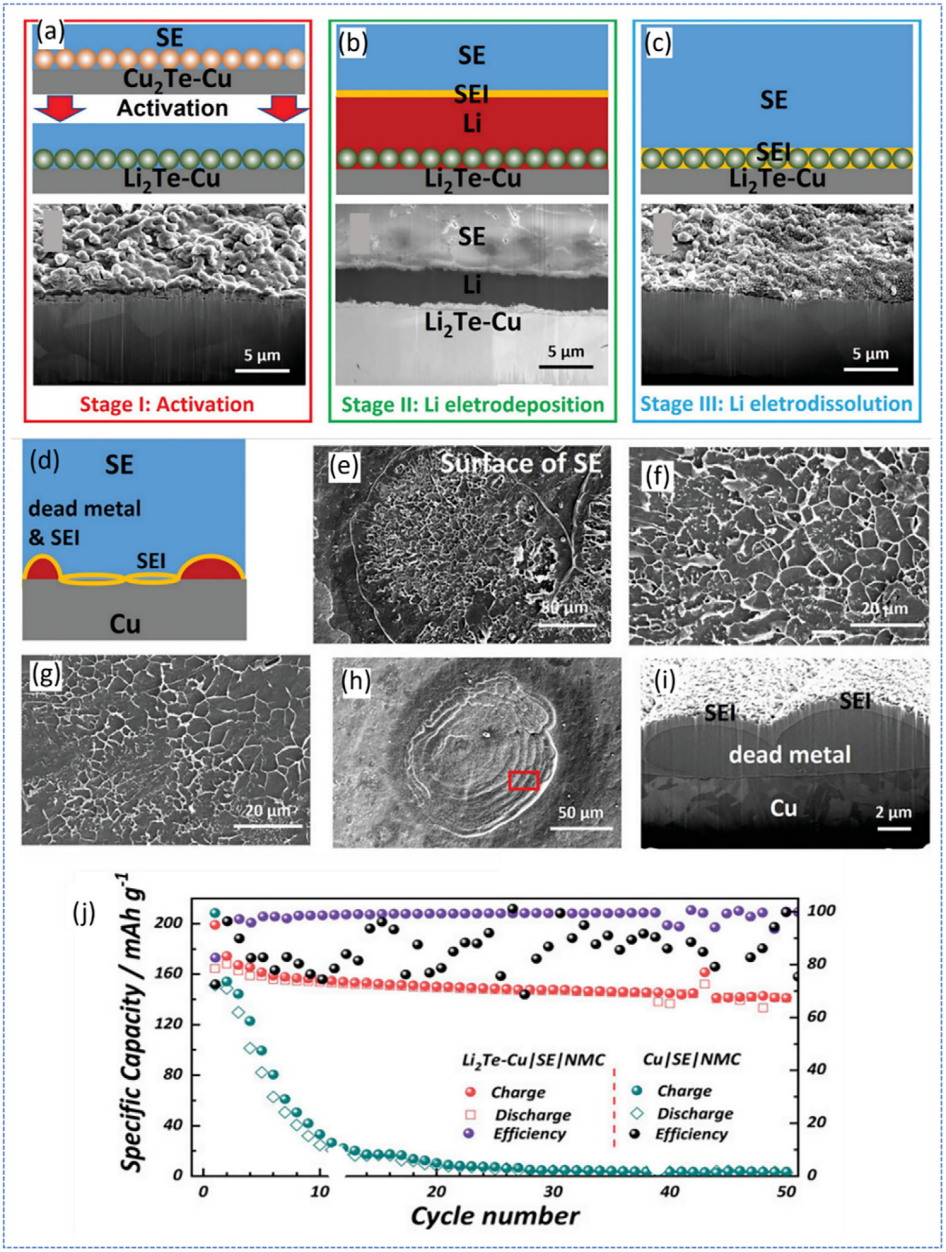
Schematic illustrations and top‐down/cross‐sectional SEM images of the Li|SE|Li_2_Te‐Cu cell after a) initial activation, b) Li plating with 1 mA h cm^−2^, and c) Li stripping up to 1.0 V. d) Schematic of the Li|SE|Cu cell after Li stripping, showing a honeycomb‐like SEI and remaining dead Li. e,f) Top‐view SEM images of the SE surface after cell separation. g) SEM image of the Cu surface from a different region. h,i) Top‐view and cryo‐FIB cross‐sectional SEM images showing inactive dead Li on the stripped surface. Adapted with permission.^[^ ^40^ ^]^ Copyright 2022, Wiley‐VCH.

In half‐cell configurations using Li_6_PS_5_Cl as the sulfide electrolyte, the Li_2_Te‐modified Cu collector demonstrated significantly lower nucleation overpotentials, enhanced Coulombic efficiency (up to 96%), and resistance to dendrite‐induced short circuits, even at current densities up to 8 mA cm^−2^. Cryogenic FIB‐SEM cross‐sectional images (**Figure** **8**a–c) revealed dense, uniform Li deposition on Li_2_Te‐Cu with minimal voids or dead metal after dissolution, in stark contrast to the baseline Cu collector, which shows dendritic growth, porous SEI layers, and electrochemically inactive residues (Figure 8d–i). Full‐cell anode‐less cells incorporating NCM811 cathodes further confirmed the effectiveness of this modification, achieving an initial CE of 83% and 99% average CE over 50 cycles (Figure 8j). The enhancement is attributed to both thermodynamic lithiophilicity and electronic conductivity of the Li_2_Te‐Cu interface. This study not only introduced a scalable, non‐vacuum method for collector modification via Te vapor treatment but also underscored the fundamental role of solid‐state wetting in anode‐less ASSB design.

In parallel with surface modifications, nanostructured interlayers have emerged as a distinct strategy to regulate Li plating dynamics in anode‐less ASSBs. Unlike surface modification strategies, nanostructured interlayers introduce a physically separate layer between the current collector and SE, providing a 3D scaffold that facilitates uniform Li deposition, buffers mechanical stress, and accommodates volume changes. This multifunctional design allows better control over Li nucleation and plating dynamics across larger interfacial areas. The primary advantage of this strategy lies in its ability to simultaneously improve Li plating uniformly, suppress dendrite formation, and enhance cycling stability. However, these interlayers typically require more complex fabrication and precise control of thickness, composition, and microstructure to avoid increased interfacial impedance or hindered Li transport.^[^ ^61^ ^]^

**Figure 9:**
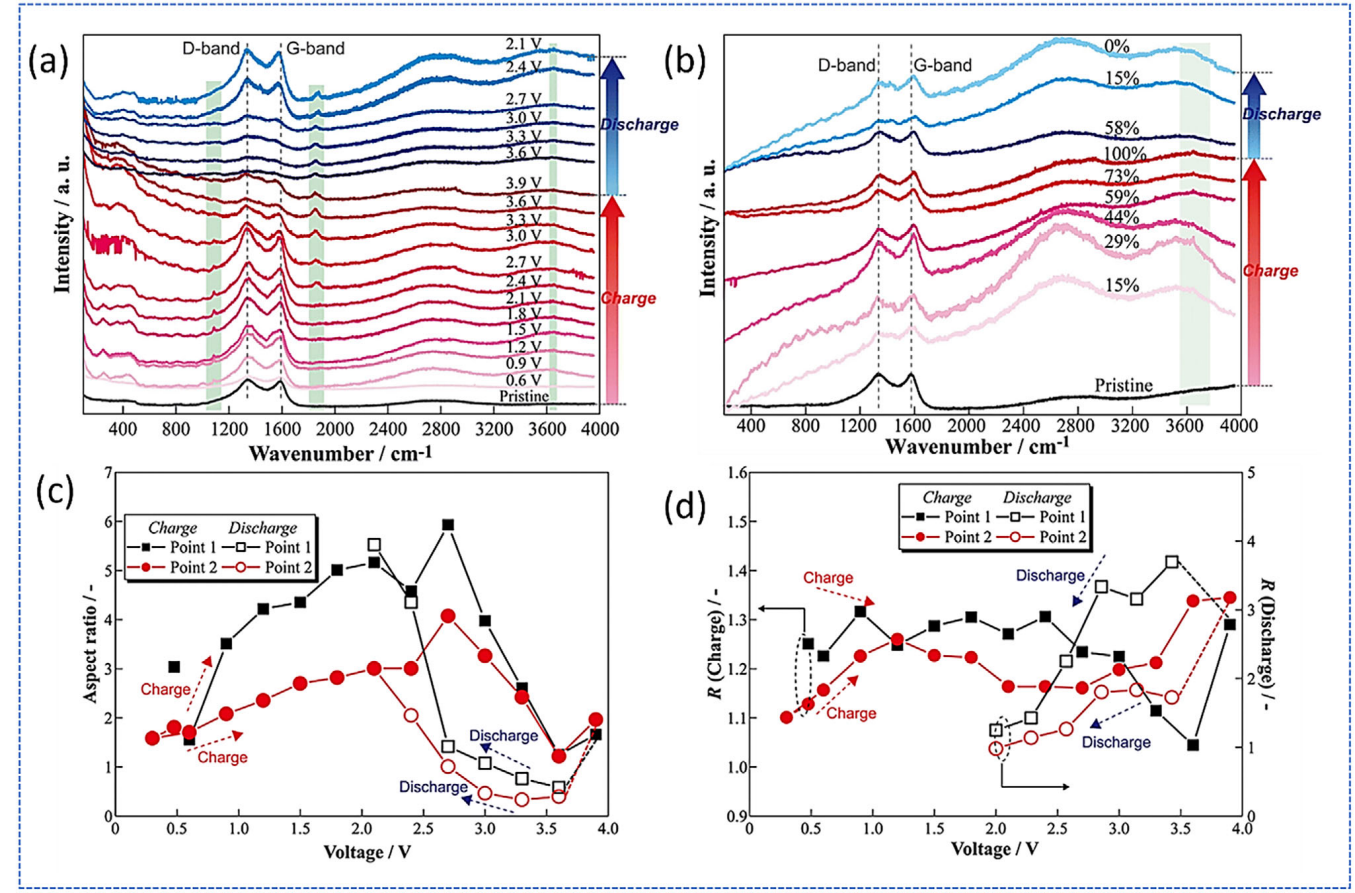
a) Raman spectra were recorded at point 1 in the Ag/C layer of the anode‐less ASSB during charging and discharging, with measurements taken every 0.3 V at room temperature. b) Raman spectra were collected from the Ag/C layer in the cross‐section of the anode‐less ASSB at each state of charge during ex situ charge–discharge testing. Relationships between the aspect ratio (c), R‐value (d), and peak positions. Adapted with permission.^[^ ^61^ ^]^ Copyright 2025, Royal Society of Chemistry.

Hiraoka et al. studied the structural and electrochemical behavior of a thin Ag/C interfacial layer in anode‐less ASSBs, focusing on its role in stabilizing Li plating and improving interfacial reversibility.^[^ ^61^ ^]^ The Ag/C layer consists of nanoscale Ag particles (≈60 nm) uniformly dispersed in amorphous carbon black (≈38 nm) and coated as a thin (≈10 µm) layer on the stainless‐steel current collector. The carbon network provides electronic conductivity and allows partial Li^+^ intercalation, while the Ag nanoparticles act as lithiophilic nucleation sites that promote uniform Li deposition and Li‐Ag alloy formation. To elucidate the dynamic processes at the Ag/C interface during cycling, the authors employed operando and ex‐situ Raman spectroscopy, offering real‐time insights into local chemical changes at the current collector interface. In their anode‐less ASSB cell configuration, the Ag/C layer was inserted between the sulfide SE (Li_6_PS_5_Cl) and a stainless‐steel current collector. Operando Raman spectroscopy revealed significant changes in the D‐ and G‐band features of carbon black during charge–discharge, including variations in peak position, intensity, and shape, indicating partial Li^+^ intercalation into the disordered carbon. These changes were most pronounced in the mid‐state‐of‐charge region (≈2.1–2.4 V), as shown in **Figure** **9**a and further quantified through aspect ratio and intensity ratio (R‐value) analysis in Figure 9c,d. Additionally, new Raman peaks emerged around 1087, 1854, and 3648 cm^−1^ during charging, likely corresponding to metastable Li‐containing species or Li‐Ag alloying phase (Figure 9a).

The operando analysis also detected a fluorescence background (particularly below 1000 cm^−1^), attributed to metallic Li or alloy formation, further confirming that Li nucleation and interfacial alloying occur dynamically. While the formation of Li‐Ag alloy and Li‐intercalated carbon can, in principle, introduce local strain due to mismatched expansion coefficients, Hiraoka et al. did not report evidence of cracking or structural degradation within the Ag/C layer. Instead, these results highlight that the composite structure accommodates Li deposition uniformly and helps suppress dendrite formation. The ex‐situ Raman spectra collected after relaxed states showed only gradual D/G‐band evolution and fewer or weaker additional peaks, underscoring the importance of capturing non‐equilibrium interfacial processes through in situ techniques (Figure 9b). Overall, Hiraoka et al. showed that a thin Ag/C interlayer in anode‐free sulfide‐based ASSBs facilitates Li alloying with Ag, partial intercalation into carbon, and uniform Li plating at the current collector. This multifunctional behavior helps suppress dendrite formation, reduce interfacial stress, and improve cycling reversibility, ultimately enhancing battery stability and performance.

Building upon this concept, Liu et al. developed an Ag‐C nanocomposite current collector to address the critical interfacial instability in anode‐less sulfide‐based ASSBs.^[^ ^62^ ^]^ The study focused on improving Li metal deposition uniformity and maintaining continuous contact between the SE and the current collector. By uniformly dispersing nanoscale Ag nanoparticles within a Super P carbon matrix, the Ag‐C composite served as both a conductive scaffold and a lithiophilic interface, promoting favorable Li nucleation and diffusion. This design achieved a significantly reduced interfacial impedance (≈10 Ω.cm^2^), enabling dense Li deposition exceeding 25 µm without short‐circuiting. Notably, the Ag‐C collector maintained uniform Li plating even at high areal capacities (≈7.0 mA h.cm^−2^), with over 200 stable cycles at 0.25 mA.cm^−2^ and an average Coulombic efficiency of ≈99.7%. The improvement was attributed to the formation of Li‐Ag alloy sites acting as energetically favorable nucleation centers, effectively suppressing dendrite formation. These results validate the nanocomposite current collector design as an effective strategy for stabilizing interfaces in anode‐less sulfide‐based ASSBs.

**Figure 10:**
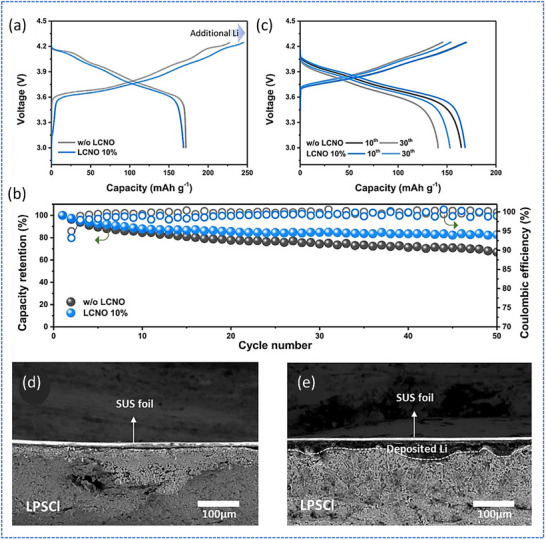
Evaluation of anode‐less sulfide‐based ASSBs (NCM811||LPSCl||SUS). a) Initial charge–discharge profiles of cells with 10% LCNO additive at 0.05 C. b) Cycling performance at 0.1 C showing capacity retention with 10% LCNO. c) Voltage profiles over multiple cycles. d) SEM image of the current collector without LCNO showing no residual Li. e) SEM image of the current collector with 10% LCNO showing Li deposition after the first cycle. Adapted with permission.^[^ ^63^ ^]^ Copyright 2023, Elsevier.

Beyond current collector engineering, active Li management via sacrificial cathode additives has proven effective in overcoming Li inventory limitations. Lee et al. explored the use of Li_2_Cu_0.6_Ni_0.4_O_2_ (LCNO) as a sacrificial cathode additive to compensate for Li loss and enhance cycling stability in anode‐less ASSBs.^[^ ^63^ ^]^ These batteries inherently lack Li on the anode side, resulting in continuous capacity fading. By incorporating LCNO into the cathode, additional Li was delivered during the initial charge, creating a more stable Li reservoir without relying on excess Li metal or pre‐lithiation. The study demonstrated that LCNO exhibited a high initial charging capacity of 393.3 mA h g^−1^, but a very low Coulombic efficiency of 5.6% in the 4.3–3.0 V window, confirming its sacrificial role. In ASSBs assembled with 10wt.% LCNO in the cathode and Li_6_PS_5_Cl (LPSCl) sulfide SE, the cells achieved a capacity retention of 82.7% over 50 cycles, a marked improvement over the control without LCNO, which showed only 67% retention (**Figure** **10**). SEM images confirmed that LCNO provided a residual Li reservoir at the current collector interface after the first cycle (Figure 10), validating its role in sustaining Li availability and interface contact. This approach effectively mitigated the challenges of Li depletion and interfacial instability in anode‐less sulfide‐based ASSBs.

Another promising approach involves engineering dual‐function interfacial coatings that stabilize both sides of the interface. Wang et al. studied the stabilization of anode‐less sulfide‐based ASSBs by engineering the current collector interface to simultaneously control two critical interphases: the Li‐SE interface (SEI‐1) and the current collector‐SE interface (SEI‐2).^[^ ^64^ ^]^ They introduced a bilayer structure composed of 140 nm Mg and 30 nm W (Mg/W) deposited onto a Cu current collector. The Mg layer promoted uniform Li electrodeposition and stripping for forming a reversible Li‐Mg alloy, while the W underlayer acted as a corrosion‐resistant barrier to prevent the formation of electronically conductive copper sulfides at the SEI‐2, which otherwise degrade electrochemical performance. Through cryo‐FIB‐SEM and EDS analysis, they demonstrated that the Mg/W‐coated current collectors retained structural integrity and prevented void formation or corrosion even after 200–300 cycles.

**Figure 11:**
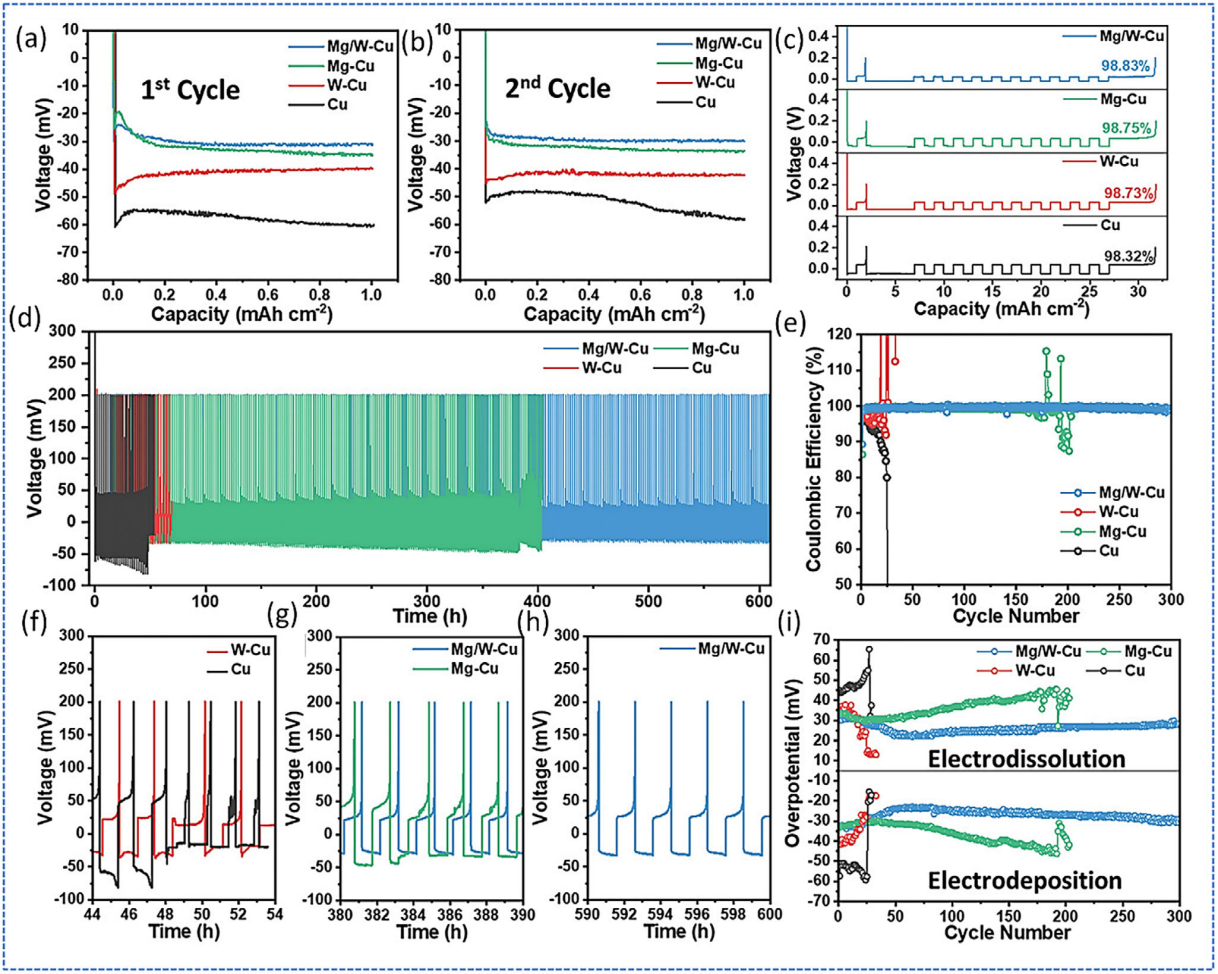
Electrochemical tests were carried out in asymmetric half‐cells using different current collectors. All cells were cycled at a current density of 1 mA cm^−2^ with a fixed Li deposited capacity of 1 mA h cm^−2^. Panels (a) and (b) show the voltage profiles during Li plating in the first and second cycles, respectively. Panel (c) presents the Coulombic efficiency (CE) of Li plating and stripping. Panels (d) and (e) display the overall cycling performance and CE over time. Panels (f) and (h) highlight selected voltage curves during cycling, and panel (i) compares the overpotentials during Li deposition and removal for the different current collectors. Adapted with permission.^[^ ^64^ ^]^ Copyright 2025, Wiley‐VCH.

**Figure** **11** compares the cycling stability and overpotentials of various current collectors, showing that the Mg/W‐Cu configuration maintained stable Coulombic efficiency and low overpotential over 300 cycles, unlike Mg‐only or W‐only coatings. The study highlighted the importance of dual‐function interfacial layers to stabilize both SEI‐1 and SEI‐2, offering a robust strategy for advancing the longevity and reliability of anode‐less sulfide‐based ASSBs.

**Figure 12:**
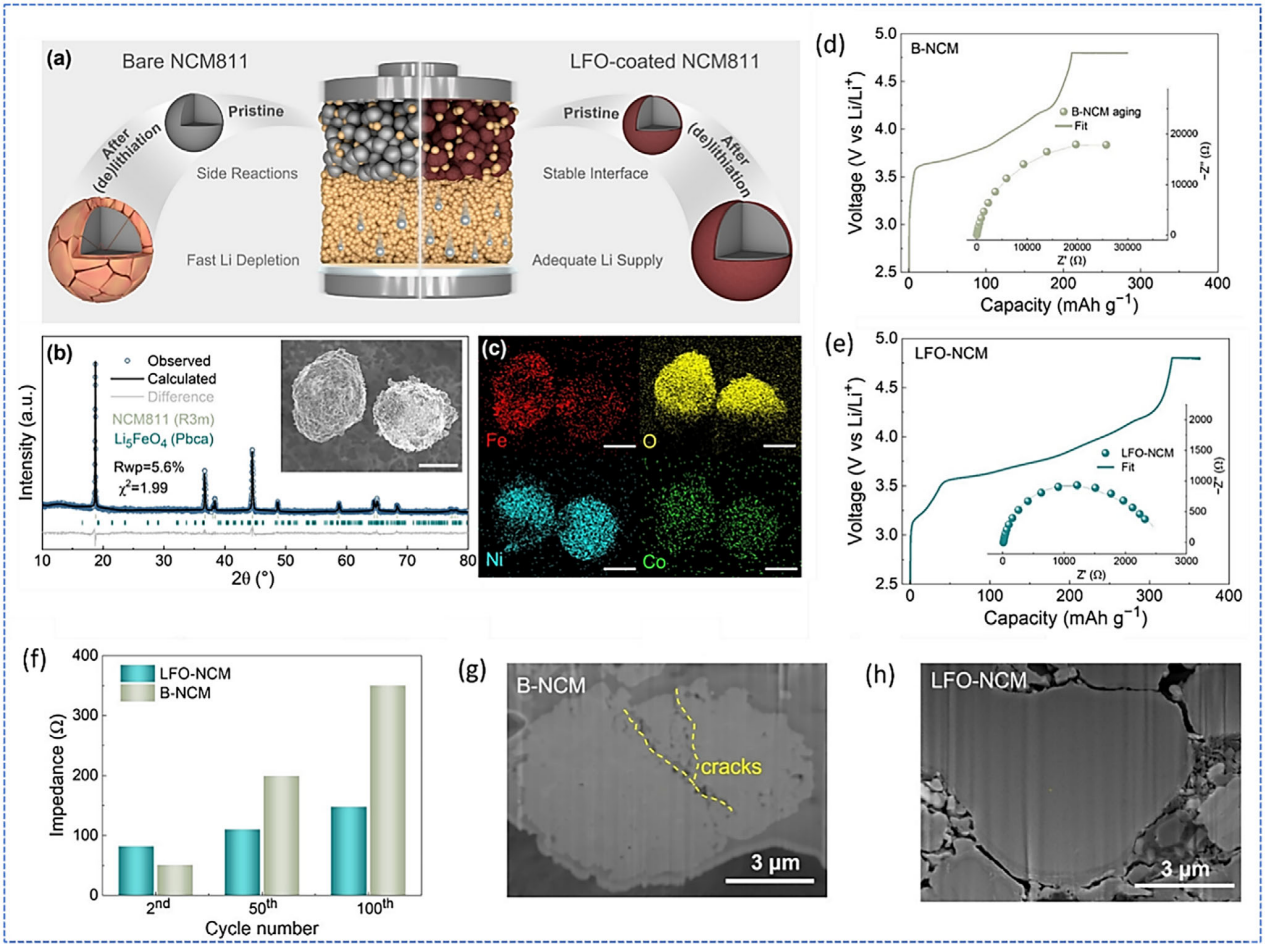
Cathode design strategy for Li‐free anode ASSBs. a) Schematic illustration of Li‐free ASSB using bare NCM811 and LFO‐coated NCM811 (LFO‐NCM) cathodes during (de)lithiation. The LFO coating serves as both a Li source and an interfacial protection layer, mitigating the decomposition of NCM811 and LPSC. b) XRD pattern of LFO‐NCM with inset SEM image. c) EDS mapping confirms the uniform distribution of LFO components on the NCM811 surface. The aging tests of d) Bare‐NCM (B‐NCM) and e) LFO‐NCM ASSBs were conducted at a constant voltage of 4.8 V after initial charging, with corresponding EIS curves (inset). f) Internal resistance of B‐NCM and LFO‐NCM after different cycles. Cross‐section SEM images of the (g) B‐NCM and (h) LFO‐NCM after 100 cycles. Adapted with permission.^[^ ^65^ ^]^ Copyright 2024, Wiley‐VCH.

To further address cathode‐side limitations, Xu et al. developed a dual‐function strategy to improve both performance and stability in Li‐free anode sulfide‐based ASSBs by integrating pre‐lithiation and interfacial protection into the cathode design.^[^ ^65^ ^]^ In their work, Li_5_FeO_4_ (LFO) was uniformly coated onto LiNi_0.8_Co_0.1_Mn_0.1_O_2_ (NCM811) cathode particles. This coating served a dual role: i) as a Li reservoir to compensate for initial losses inherent to Li‐free configurations, and ii) as a protective interlayer that suppresses detrimental reactions between the highly oxidizing NCM cathode and the sulfide SE (Li_6_PS_5_Cl, LPSC). First‐principles calculations demonstrated that the LFO/LPSC interface exhibits significantly lower reaction energies compared to the NCM811/LPSC interface, indicating improved thermodynamic compatibility. These computational results were corroborated by electrochemical impedance spectroscopy, which revealed reduced interfacial resistance, and by in situ pressure measurements, demonstrating improved mechanical integrity during cycling. Electrochemically, the use of LFO‐coated NCM811 (LFO‐NCM) cathode in Li‐free ASSBs resulted in a notable improvement in performance metrics. Compared to cells with uncoated NCM811, those with LFO‐NCM exhibited a higher reversible capacity (199.7 vs. 174.7 mA h g‐1) and significantly better capacity retention (84.8% vs. 33.8% after 100 cycles). This performance was sustained even under high‐voltage operation (up to 4.5 V) and high‐loading conditions. Importantly, the authors utilized Li‐free anode configurations, including Li‐free indium and Li‐free silicon anodes, thereby demonstrating the viability of their strategy in practical battery architectures. As illustrated in **Figure** **12**a–c, the schematic and material characterization confirmed the uniform LFO coating, while Figure 12d–h highlighted the reduced impedance growth and suppression of cathode degradation over extended cycling. Collectively, this work presents a scalable and effective approach to stabilizing Li‐free anode sulfide‐based ASSBs through cathode‐side engineering that simultaneously addresses Li inventory and interfacial instability challenges.

**Figure 13:**
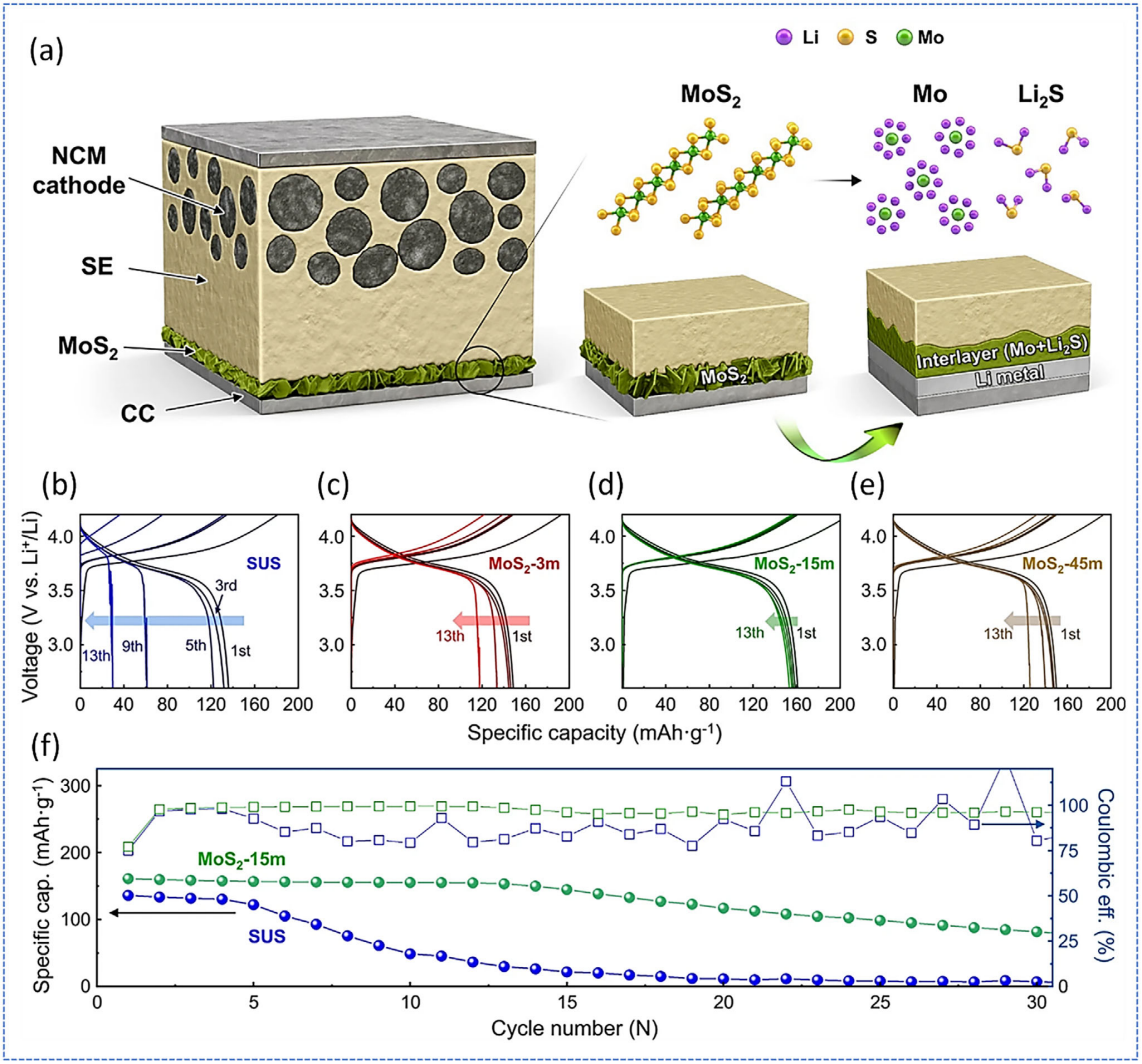
a) Schematic illustration of the Li‐free full‐cell architecture employing an NCM cathode and MoS_2_‐modified current collector, highlighting the interfacial conversion of MoS_2_ into a Mo + Li_2_S interlayer during lithiation. b–e) Galvanostatic charge–discharge curves of full‐cells using different current collectors: b) bare SUS, c) MoS_2_ grown for 3 min, d) MoS_2_ grown for 15 min, and e) MoS_2_ grown for 45 min. f) Comparative cycling performance and coulombic efficiency of the SUS and MoS_2_‐15 min full cells at 0.2C. Adapted under the terms of the CC‐BY 4.0 license.^[^ ^66^ ^]^ Copyright 2025, The Author(s).

Finally, interfacial chemistry optimization using sacrificial interlayers also presents a promising direction. Seo et al. proposed a strategy to stabilize Li‐free ASSBs by engineering the anode‐electrolyte interface using a sacrificial MoS_2_ thin film.^[^ ^66^ ^]^ In these Li‐free full cells, Li is sourced entirely from the cathode (LiNi_0.6_Co_0.2_Mn_0.2_O_2_), while the anode consists of a stainless steel (SUS) current collector modified with a thin MoS_2_ coating. Upon initial lithiation, the MoS_2_ undergoes a conversion reaction to form an interlayer composed of Mo metal and Li_2_S, which promotes uniform Li nucleation and suppresses dendritic growth. This artificial solid electrolyte interphase (ASEI) effectively reduces nucleation overpotential and enhances interfacial stability between the current collector and sulfide SE (Li_6_PS_5_Cl), resulting in significantly improved electrochemical performance in full cells. A key highlight of this work is shown in **Figure** **13**, which illustrates the structure and interface design of the MoS_2_‐modified Li‐free full cell. Compared to the bare SUS control (Figure 13b), the MoS_2_‐coated cells (Figure 13c–e) displayed higher initial discharge capacities and markedly improved cycling stability. Notably, the MoS_2_‐15 min coating (Figure 13d) achieved the best performance, delivering an initial discharge capacity of 161.1 mA h g^−1^ and maintaining 58.9% capacity retention after 20 cycles, where the SUS‐based cell retained only 8.3% (Figure 13f). This enhancement was attributed to optimized interfacial properties and reduced charge transfer resistance. The study confirms that controlling the thickness and morphology of MoS_2_ is critical; overly thick layers (e.g., MoS_2_‐45 min) increased impedance due to excessive SEI formation, while thinner films (MoS_2_‐3 min) were insufficient to fully passivate the interface. Overall, this work demonstrates a practical and scalable approach to stabilize anode‐less (Li‐free) sulfide‐based ASSBs through the rational design of interfacial chemistry, offering a path toward safer and higher energy solid‐state battery systems.

In addition to such interfacial engineering approaches, recent advances in solid electrolytes have also demonstrated performance improvements through halogenation and anode interface modification, further highlighting diverse pathways for stabilizing ASSBs.^[^ ^67^, ^68^, ^69^ ^]^

Together, these studies demonstrate the effectiveness of the four main approaches‐ current collector engineering, interlayer design, cathode prelithiation, and SEI modification‐ in mitigating interfacial degradation in anode‐less sulfide‐based ASSBs. Each strategy targets particular failure modes, and **Table** **1** provides a side‐by‐side comparison of their key function, advantages, and limitations. While each approach improves certain aspects of interfacial stability, combining them is essential to achieve stable, high‐performance, and commercially viable sulfide‐based ASSBs.

**Table 1:** Comparison of strategies to enhance interfacial stability in anode‐free sulfide‐based ASSBs, highlighting their advantages and limitations.

Strategy	Key Function	Advantages	Limitations	Refs.
Current collector engineering	Promotes uniform Li nucleation	Suppresses dendrite growth, improves cycling stability	Scalability concerns, fabrication complexity	[40, 70]
Interlayer design	Provides chemical and mechanical stabilization at the Li/SE interface	Reduces electrolyte decomposition, enhances interfacial contact	Additional processing steps, added materials, and potential interface mismatch	[61, 71]
Cathode prelithiation	Compensates for intrinsic Li loss	Improves initial capacity, reduces voltage drop	Additional cost, manufacturing steps, and handling safety issues	[65, 72]
Interphase modification	Enhances interfacial stability	Suppresses decomposition, improves Li^+^ transport	Synthetic challenges, limited material availability	[66, 73]

## 4 Summary and Future Prospects

Anode‐less sulfide‐based ASSBs represent a transformative class of energy storage devices, offering the potential to enhance safety, energy density, and manufacturing simplicity. By eliminating excess Li metal and relying solely on Li extracted from the cathode during the initial charge, these systems achieve a lean, high‐energy design. However, the absence of Li at the anode introduces several challenges, including unstable Li nucleation, rapid interfacial degradation, void formation during stripping, and poor Coulombic efficiency, particularly in contact with reactive sulfide‐based SEs. To overcome these limitations, a variety of strategies have been explored, ranging from interfacial engineering and cathode prelithiation to the development of artificial solid electrolyte interphases (ASEIs). Collectively, these approaches have demonstrated notable improvements in capacity retention, interfacial stability, and cycling reversibility in Li‐free solid‐state systems.

Looking forward, several key areas of future research and development must be prioritized to transition AF‐ASSBs from laboratory concepts to viable commercial products.

### 4.1 Advanced Current Collector Engineering

The choice and surface design of current collectors play a decisive role in directing Li nucleation and preventing inhomogeneous deposition. Future work should move beyond inert metallic foils (e.g., Cu, SUS) toward lithophilic and textured current collectors, possibly incorporating nanoscale surface features or lithophilic seed layers (e.g., Ag, Zn, Sn‐based coatings) that can lower nucleation barriers. Scalable deposition methods such as physical vapor deposition (PVD), electrodeposition, or atomic layer deposition (ALD) may be integrated into roll‐to‐roll processes to maintain compatibility with manufacturing standards. Moreover, designing 3D current collectors with engineered porosity or vertical ion channels may help buffer volume fluctuations and facilitate uniform Li growth.

### 4.2 Rational Design of Interlayers and Artificial Interfaces

A critical priority is the development of multifunctional interlayers that can enhance ionic conductivity, suppress side reactions, and buffer mechanical stresses at the Li/SE interface. Promising candidates include redox‐active transition metal sulfides (e.g., MoS_2_, TiS_2_), polymer‐ceramic hybrids, and Li‐containing alloys that transform in situ to form conductive and chemically compatible interfaces. Future designs should also account for mechanical compliance, ensuring that interlayers can accommodate Li volume changes without delamination or fracture. Additionally, ASEIs formed via sacrificial films or in situ chemical reactions show promise; however, their long‐term stability under lean Li conditions remains a key area for further investigation.

### 4.3 Cathode‐Side Li Compensation and Prelithiation Techniques

Since anode‐less ASSBs begin with zero‐excess Li, efficient Li management on the cathode side becomes essential. Prelithiation strategies using sacrificial Li‐containing compounds (e.g., Li_5_FeO_4_, Li_2_O_2_, stabilized Li metal powder) or surface‐coated Li reservoirs must be further optimized for compatibility with sulfide electrolytes. Importantly, these materials must not only offer high Li yield but also exhibit minimal parasitic reactivity with cathodes and sulfide SEs. Future research should also explore dynamic Li balancing during cycling and investigate the possibility of self‐healing or replenishing interlayers.

### 4.4 Fundamental Characterization and Diagnostic Techniques

Advancing anode‐less ASSBs will require a deeper mechanistic understanding of Li nucleation, interface degradation, and mechanical stress evolution during cycling. Recent in situ and operando methods have been developed to address these challenges. For example, operando X‐ray tomography has been used to track void formation and dendrite propagation at buried Li/SE interfaces,^[^ ^53^, ^74^ ^]^ while cryo‐TEM provides atomic‐scale insights into Li nucleation and ASEI formation under reactive sulfide environments.^[^ ^75^ ^]^ Neutron depth profiling enables non‐destructive quantification of Li distribution across layers,^[^ ^76^ ^]^ and synchrotron‐based XRD, XPS, and XAS have revealed interfacial phase evolution and redox reactions in real time.^[^ ^57^, ^77^ ^]^ In addition, acoustic emission techniques^[^ ^78^ ^]^ and in situ stress sensors have been employed to monitor mechanical degradation, whereas electrochemical strain microscopy (ESM) allows nanoscale mapping of Li‐ion transport and interface dynamics.^[^ ^79^ ^]^ Together, these complementary approaches provide a powerful toolbox to unravel the complex electro‐chemo‐mechanical processes in anode‐less sulfide‐based ASSBs. However, further development of multimodal operando platforms will be critical to capture the coupled processes that ultimately govern cycling stability.

### 4.5 Mechanical and Processing Challenges in Scalable Architectures

The pressure‐sensitive nature of sulfide SEs poses a dilemma: sufficient stack pressure is needed to maintain interfacial contact, yet excessive pressure is impractical for large‐scale manufacturing and raises concerns for pouch cell integration. Research should focus on developing compliant interlayers and self‐adaptive architectures that allow operation at low to moderate pressure (<5 MPa). Additionally, fabrication methods such as cold sintering, dry coating, and low‐temperature densification must be refined to accommodate the thermal and chemical sensitivity of sulfide systems. Achieving high cathode loadings (>4 mA h cm^−2^) while maintaining interfacial integrity will be essential to realize competitive energy densities.

### 4.6 Full‐Cell Integration and Realistic Testing Protocols

Bridging the gap between academic research and industrial implementation requires rigorous validation of full‐cell configurations under practical conditions. Key considerations include high‐loading electrodes, lean electrolyte usage, wide temperature operation, and realistic charge/discharge rates. Long‐term cycling (>500 cycles), calendar aging, and abuse tolerance should also be assessed to ensure commercial relevance. Additionally, system‐level metrics such as Li inventory efficiency, stack‐level energy density (Wh L^−1^), and cost‐per‐kWh must be integrated into evaluation frameworks to provide a comprehensive understanding of performance and feasibility.

In conclusion, the advancement of anode‐less sulfide‐based ASSBs will hinge on holistic interface engineering, intelligent materials design, and manufacturing innovation. While significant strides have been made in addressing the initial challenges, continued interdisciplinary efforts across materials science, electrochemistry, mechanics, and process engineering are essential to unlock their full potential. With coordinated progress across these fronts, anode‐less ASSBs are poised to emerge as a disruptive force in the pursuit of safe, high‐performance, and sustainable energy storage systems.

## Conflict of Interest

The authors declare no conflict of interest.
